# Why?

**Published:** 1870-10

**Authors:** 


BY UNCLE BOB.

Looking over, in my mind, a variety of *home* questions or subjects for a talk with "the family," I find no one more important than this one of "why."

I wish to speak of a very common and very unpleasant fault prevailing in many families.

Every command or refusal to the younger members of it, is immediately submitted to the test question of "why," or "why can't I."

To an observant person who is thrown in contact with a family which is blessed with a number of children, there is no quicker expose of its internal management and discipline than the manner in which this little monosyllable is met and disposed of.

Let Tommy be refused any desire, and the extent of his government, or his parents, is instantly shown by the way which he sways this questioning word. "*Why*" comes ringing out, or is peevishly whined, or more likely is deprecatingly snarled, if he has been allowed to question the authority of his parent. The little emperor makes his attack upon the household head, and perhaps carries the day with but a single volley from this effective weapon. Or if his attack is met with but a half resistance, he is sure of his success if he continues the assault, reinforced with a little show of temper and a few whines in his tones. If he dislikes any command, he must immediately know the reason *why* it is given. If he is refused a request, he must know *why* it is denied. In short, no authority is acknowledged by him which is not submissive to his imperious *why*.

This habit is allowed to commence before the word can be distinctly spoken. Gradually the parent is drawn into explaining the reason of his or her denial.

As a general thing, a parent or any one using authority should not admit an inquisitive comment upon immediate rulings. Obedience should be first enforced. Do not refuse unless you mean what you say. A doubtful refusal is as bad in its result as entire neglect. You indicate strength of authority with a lack of wisdom to decide.

Authority exercised upon children is not to be questioned by them as equals. This is the vulnerable po[illegible]t at which to att[illegible]k the question of *why*. The [illegible]rent's c[illegible] man[illegible] is a rule plainly to be obeyed because it is a parent's. Consider the relative age and comparative experience of a child of three or four years and the mother. Is it necessary to explain the absurdity of compelling the "reason why" to be given when the mother says "no?"

This last remark I do not intend shall be taken as a wholesale relief from judicious explanations of the parent's wishes. A true-hearted and loving mother, at reasonable times, will delight to take her child up into her lap, and by a skillful story or apt illustration, clearly show the little one that because she is a *mother* she is the best judge of the proper rule to give.

The infallibility of "father" and "mother" is a matter which must be very early established in a child's mind. I love to hear any child tell his playmate in a disputed point in their play that his papa told him so and "he knows that *he* knows." "My child it is sufficient that I tell you so," is reason enough for any boy or girl who has been rightly trained.

The precision and beauty of a military movement is a sight which thrills every one who witnesses it. Who questions the discipline which makes order after order so quietly and quickly obeyed? Let the commanding officer be clear headed and his company will bring him and itself praise for its unquestioning obedience.

I fear that while good parents accept the reasoning of this, that another portion who cannot be blessed as such, may shield themselves behind these last remarks and cause the whims of their ignorance to stand sheltered from criticism.

To all I say:—be sure you know "the *why*" yourself, otherwise you stultify yourself. Your child may win credit for obeying you while you deserve censure for a foolish and perhaps hasty crossings of its wish. It is a matter which should occupy much study and positive care—the checking of the childish plans. Do not stunt independency of thought, but rather guide it. Lead your child to exercise good judgment in every manner.

There never ought to be any needless indetermination in your own decision. Tell your child if you cannot make him understand, that he must acknowledge your judgment, be happy and be content. You need not check his self-reasoning, but you must ensure his obedience.—The "*why*" will then cease to become a formidable weapon against you. A happy family will have rewarded your honest attempts to lead it up to a skillful exercise of self-reliance and cheerful obedience. You will hardly notice your *authority* as being such, but have the pleasure of knowing that the affection of your family is binding them to you while you are carefully relaxing your authority over it.

Let me say in conclusion, beware of the little word *why* when exercising your judgment in conflict with your children.

